# Adjunctive Zoledronate + IL-2 administrations enhance anti-tuberculosis Vγ2Vδ2 T-effector populations, and improve treatment outcome of multidrug-resistant tuberculosis^1^

**DOI:** 10.1080/22221751.2022.2095930

**Published:** 2022-07-21

**Authors:** Hongbo Shen, Enzhuo Yang, Ming Guo, Rui Yang, Guixian Huang, Ying Peng, Wei Sha, Feifei Wang, Ling Shen

**Affiliations:** a Shanghai Clinical Research Center for Infectious Disease (tuberculosis), Shanghai Institute of Infectious Disease and Biosecurity, Shanghai Key Laboratory of Tuberculosis, Shanghai Pulmonary Hospital, Institute for Advanced Study, Tongji University School of Medicine, Shanghai, People’s Republic of China; b Department of Microbiology & Immunology and Center for Primate Biomedical Research, University of Illinois College of Medicine, Chicago, IL, USA; c State Key Laboratory of Virology, Modern Virology Research Center, College of Life Sciences, Wuhan University, Wuhan, People’s Republic of China; d Department of Medical Microbiology and Parasitology, Key Laboratory of Medical Molecular Virology (MOE/NHC/CAMS), Shanghai Institute of Infectious Disease and Biosecurity, School of Basic Medical Sciences, Shanghai Medical College, Fudan University, Shanghai, People’s Republic of China

**Keywords:** Multidrug-resistant tuberculosis, adjunctive immunotherapy, Vγ2Vδ2 T cells, nonhuman primates, Zoledronate, IL-2

## Abstract

Multidrug-resistant tuberculosis (MDR-TB) is a refractory disease with high mortality rate due to no or few choices of antibiotics. Adjunctive immunotherapy may help improve treatment outcome of MDR-TB. Our decade-long studies demonstrated that phosphoantigen-specific Vγ2Vδ2 T cells play protective roles in immunity against TB. Here, we hypothesized that enhancing protective Vγ2Vδ2 T-effector cells could improve treatment outcome of MDR-TB. To address this, we employed clinically approved drugs Zoledronate (ZOL) and IL-2 to induce anti-TB Vγ2Vδ2 T-effector cells as adjunctive immunotherapy against MDR-TB infection of macaques. We found that adjunctive ZOL/IL-2 administrations during TB drugs treatment of MDR-TB-infected macaques significantly expanded Vγ2Vδ2 T cells and enhanced/sustained Vγ2Vδ2 T-effector subpopulation producing anti-TB cytokines until week 21. ZOL/IL-2 administrations, while expanding Vγ2Vδ2 T cells, significantly increased/sustained numbers of circulating CD4^+^ Th1 and CD8^+^ Th1-like effector populations, with some γδ T- or αβ T-effector populations trafficking to airway at week 3 until week 19 or 21 after MDR-TB infection. Adjunctive ZOL/IL-2 administrations after MDR-TB infection led to lower bacterial burdens in lungs than TB drugs alone, IL-2 alone or saline controls, and resulted in milder MDR-TB pathology/lesions. Thus, adjunctive Zoledronate + IL-2 administrations can enhance anti-TB Vγ2Vδ2 T- and αβ T-effector populations, and improve treatment outcome of MDR-TB.

## Introduction

Multidrug-resistant tuberculosis (MDR-TB) mostly evolves from failures in treatments of chronic *Mycobacteria tuberculosis* (Mtb) infection. MDR-TB is defined by the combined resistance to at least two of the most powerful first-line anti-TB drugs of isoniazid and rifampin, and remains a global public health crisis, with 77,000 new cases in 2019 [1]. MDR-TB has a >50% mortality rate because of no or few options of drugs. Second-line anti-TB drugs cost more, require longer treatment, and have more adverse effects[2]. So it is urgent to develop novel host-directed therapy (HDT) in combination with TB antibiotics treatment of MDR-TB. Such adjunctive HDT or immunotherapeutic would provide prospects of shortening treatment duration, improving treatment outcomes, and preventing lung damage [3]. The fundamental elements in HDT for TB are to identify the effective immune regimen targeting immune components of anti-TB immunity [4].

The decades-long studies from us and others demonstrate that Vγ2Vδ2 T cells specific for phosphoantigen including isopentenyl pyrophosphate (IPP) exist only in humans and nonhuman primates (NHP), represent a highly-dominant circulating γδ T cell subpopulation and play protective roles in immunity against TB and other infections [5,6]. We and others have also established unique *in vivo* manipulating systems including Zoledronate (ZOL) plus interleukin (IL)-2 to remarkably active and expand Vγ2Vδ2 T cells for immune interventions against TB infections or cancers [7,8]. ZOL and IL-2 are clinically approved drugs for the treatment of osteoporosis and other diseases. ZOL has been employed for γδ T-cell targeted cancer therapy since it could provoke accumulation of the intermediate metabolite IPP causing activation of human Vγ2Vδ2 T cells [9].

To explore adjunctive HDT or immunotherapeutics against MDR-TB, we have recently developed the first NHP model of MDR-TB infection [10]. In the current study, we employed adjunctive ZOL/IL-2 administrations in combination of TB drugs treatment of MDR-TB-infected NHP, and determined whether such adjunctive ZOL/IL-2 regimen could activate/differentiate protective Vγ2Vδ2 T effector cells and provide immunotherapeutic against MDR-TB in NHP.

## Materials and methods

### Strains, animals, infection, and treatment

Clinical isolated *M. tuberculosis* V791 strain was provided by ABSL-III lab of Wuhan University and it was proved to be MDR-TB strain (MDR-Mtb) since it is at least resistant to rifampicin (RIF), isoniazid (NIH), ofloxacin (OFX) and streptomycin (SM) as described in our previous study[10,11].

**Figure 1.**
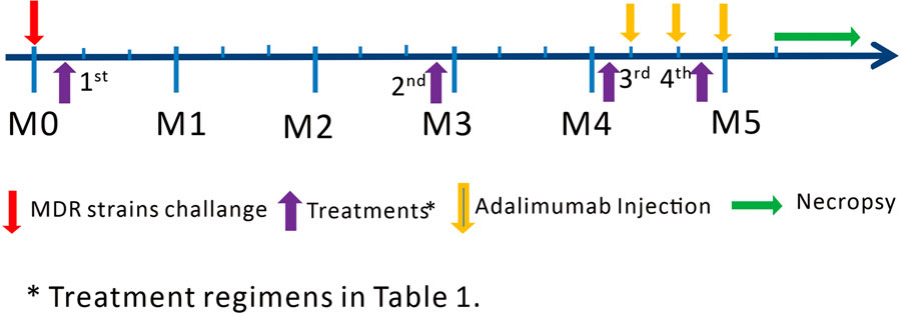
Experiment Schema. Monkeys in four groups were taken 4 times of treatment according to Table 1 since one week after MDR-Mtb strain V791 challenge, respectively.

Four- to six-year-old Chinese cynomolgus macaques with 3–5 kg body weights were used in this study. Twenty-four macaques were infected with MDR-Mtb V791 via bronchoscope-guided spread of 500 CFU into the right caudal lung lobe. Infected-macaques were divided into four groups, and were treated according to the schedules starting 7 days after MDR-Mtb V791 infection (Figure 1, Table 1, and Supplemental Table). Macaques in Group 1 is treated with anti-TB drugs; macaques in Group 2 were treated with anti-TB drugs plus Zoledronic acid (Zol) and interleukin-2 (IL-2) (one was early moribund due to severe anaphylaxis to ZOL/IL2 treatment, without endpoint bacilli counts); macaques in Group 3 were treated with anti-TB drugs plus IL-2; macaques in Group 4 were treated with saline as control. We adopted intermittent treatments with TB drugs, with short-term intervals (without TB drugs) in 12-day cycles (Table 1). Such short-term omission of TB drugs before the next treatment cycle presumably would result in a low or residual level of MDR-Mtb infection. Such a low/residual infection setting would make it more readily to determine whether adjunctive ZOL/IL-2 or IL-2 regimen could exert better therapeutic effects than TB drugs alone. This would also avoid the potential possibility that TB drugs overshadow or cover potential immunotherapeutic effects (see more in Results/Discussion) [12].

**Table 1.** Treatments.

Groups	1st treatment	2nd treatment	3rd treatment	4th treatment
Group 1	2 doses of E at 1st and 7th day;	2 doses of E at 1st and 7th day;	1 dose of E;	1 dose of E;
	2 doses of Mfx at 1st and 7th day;	2 doses of Mfx at 1st and 7th day;	1 dose of Mfx;	1 dose of Mfx;
	2 doses of CM at 1st and 7th day;	2 doses of CM at 1st and 7th day;	1 dose of CM;	1 dose of CM;
Group 2	2 doses of E at 1st and 7th day;	2 doses of E at 1st and 7th day;	1 dose of E;	1 dose of E;
	2 doses of Mfx at 1st and 7th day;	2 doses of Mfx at 1st and 7th day	1 dose of Mfx;	1 dose of Mfx;
	2 doses of CM at 1st and 7th day;	2 doses of CM at 1st and 7th day;	1 dose of CM;	1 dose of CM
	10 doses of IL-2 at 1–5 and 7–11days;	10 doses of IL-2 at 1–5 and 7–11 days;	5 doses of IL-2;	5 doses of IL-2;
	2 doses of ZOL at 1st and 7th day;	2 doses of ZOL at 1st and 7th day;	1 dose of ZOL;	1 dose of ZOL;
Group 3	2 doses of E at 1st and 7th day;	2 doses of E at 1st and 7th day;	1 dose of E;	1 dose of E;
	2 doses of Mfx at 1st and 7th day;	2 doses of Mfx at 1st and 7th day;	1 dose of Mfx;	1 dose of Mfx;
	2 doses of CM at 1st and 7th day;	2 doses of CM at 1st and 7th day;	1 dose of CM;	1 dose of CM;
	10 doses of IL-2 at 1–5 and 7–11 days;	10 doses of IL-2 at 1–5 and 7–11 days;	5 doses of IL-2;	5 doses of IL-2;
Group 4	10 doses of sterile saline at 1–5 and 7–11 days	10 doses of sterile saline at 1–5 and 7–11 days	5 doses of sterile saline	5 doses of sterile saline

To optimally uncover potential subclinical/inactive MDR-Mtb V791 infection in drug-treated animals, each macaque in four studied groups was injected subcutaneously with Adalimumab (ABBOTT, IL, USA) at the dose of 2.5 mg/kg following the ABBOTT’s instruction during the last 4 weeks before the endpoint, as described previously in nonhuman TB studies [13]. Such unbiased Adalimumab treatment allowed for enhancing and activating any inactive or subclinical MDR-TB infection that had been potentially contained through ∼4 months of intermittent TB drug treatments and immune-based interventions.

Blood and broncho-alveolar lavage (BAL) fluid were collected from these macaques after infection according to our previously described [10,14]. All animals were maintained and used at the Wuhan ABSL-III facilities in accordance with guidelines of the institutional animal care and use committee, and experimental procedures were approved by the institutional biohazard committee. Animals were anaesthetized with 10 mg/kg Ketamine HCl (Fort Dodge animal health, Fort Dodge, IA) i.m. for blood sampling. EDTA-anti-coagulated blood was collected for lymphocytes separation. For BAL sampling, after overnight fasting animals were tranquilized i.m. with 1–2 mg/kg xylazine (Ben Venue Laboratories, Bedford, OH) and 10 mg/kg ketamine HCl, and then given 0.05 mg/kg atropine (Phoenix Scientific, Inc., St. Joseph, MO) i.m. as an anticholinergic for BAL while being restrained in an upright position.

### Isolation of lymphocytes from blood and BAL fluid

These were done exactly the same as our described before [15]. Briefly, PBMC were isolated from freshly collected EDTA blood by Ficoll-Paqueplus (Amersham, NJ) density gradient centrifugation. Fresh BAL fluid was filtered through 40-µm cell strainers (BD) followed by 5 min × 1500 rpm centrifugation. Cell pellets were treated with 5 mL RBC blood lysis buffer (Sigma-Aldrich) for 10 min or waited till the suspension became clear and washed once with 5% FBS-PBS (FBS, Fetal Bovine Serum; PBS, phosphate buffer saline).

### Mycobacteria strains and cell culture

The clinical isolated *M. tuberculosis* V791 strain [10], *M. bovis* Bacillus Calmette-Guerin (BCG) Danish strain (ATCC35733) were grown at 37°C in Difco Middlebrook 7H9 broth or on Middlebrook 7H10 agar (Becton Dickinson) supplemented with 10% oleic acid-albumin-dextrose-catalase-enriched Middlebrook (OADC, Becton Dickinson), 0.2% glycerol and 0.05% Tween-80.

Isolated lymphocytes from macaque blood and BAL fluid, and the human alveolar epithelial cell line A549 were grown in RPMI 1640 medium, supplemented with 50 U/mL of penicillin and 50 µg/mL of streptomycin, 10% heat-inactivated FBS.

Cells from PBMC or BAL (1 × 10^6^/mL) were cultured in 96-U-well plates in the absence or presence of ZOL (Sigma-Aldrich) at 5 µg/mL or HMBPP ((E)-4-hydroxy-3-methyl-but-enyl pyrophosphate, Sigma-Aldrich) at 50 ng/mL, and then supplemented at day 0, 3, and 5 with 20 U/mL IL-2 (Sigma-Aldrich)[16]. On day 7, cells were harvested for surface or intracellular cytokine staining and flow cytometry analyses, or were used to isolate Vγ2Vδ2 T cells.

### Intracellular *Mycobacterial* growth inhibition assay

*M.bovis* BCG-infected A549 cells were prepared as target cells at MOI = 10 as we previously described [17,18]. To isolate Vγ2Vδ2 T cells, macaque PBMCs in ZOL + IL-2 cultures were stained with PE-anti-Vδ2 (B6, Biolegend), and then incubated with anti-PE microbeads (Miltenyi Biotech). Vδ2^+^ T cells were then isolated using MACS Separation columns (Miltenyi Biotech) according to manufacturer’s protocol, serving as effector cells. The B cells were isolated from PBMCs with CD19^+^ B cells isolation kit (Miltenyi Biotech) according manufacturer’s protocol. BCG-infected A549 cells were cultured with media alone or with purified Vδ2^+^ or CD19^+^ cells at a ratio of E: T = 10: 1 in 96-well plates for 3 days. *Mycobacteria* viability was quantified via counting CFU as previously described [19].

### Flow cytometry analysis and antibodies

The following Abs were used for culture or surface and intracellular cytokine staining for flow cytometry: CD28 (CD28.2, BD), CD49d (9F10, BD), CD3-Pacific blue (SP34-2,BD), CD4-BV510 (L200, BD), CD8- Pacific blue (RPA-T8, BD), IFN-γ-APC (4S.B3, BD), IFN-γ Brilliant Violet 711 (4S.B3, Biolegend), TNF-α-PE (Mab11, BD), TNF-α-PE-Cy7 (Mab11, BD), IL-17-PE (eBio64CAP17, eBioscience), IL-22-biotinylated (anti-human IL-22, RD), Streptavidin-Pacific blue (invitrogen), Perforin-biotinylated (Pf-344, Mabtech), Caspase 3-AF647 (C92-605,BD), anti-Vγ2-FITC (7A5, Pierce).

After staining, cells were fixed and subjected to analysis on flow cytometer of BD LSRFortessa^TM^ Cell Analyzer. Lymphocytes were gated based on forward- and side-scatters, and at least 40,000 gated events were analyzed using Summit Data Acquisition and Analysis Software (Dako Cytomation).

### Direct intracellular cytokine staining (ICS) and conventional ICS assays

Direct ICS was done exactly the same as we previously described [16]. Briefly, PBMC or lymphocytes from BAL fluid were used in each reaction to measure T cells and CD3-negative lymphocytes that could constitutively produce IFN-γ, TNF-α, IL-17, IL-22, and perforin without Ag stimulation *in vitro*. Lymphocytes were incubated for one hour with medium in presence of CD28 (1 µg/mL) and CD49d (1 µg/mL) mAbs in a 200 µL final volume at 37°C, 5% CO_2_, followed by five-hour incubation in the presence of brefeldin A (GolgiPlug, BD). After a total of six-hour incubation, cells were for surface and intracellular staining.

For conventional ICS assay, antigen PPD or HMBPP was added to the culture and incubated for 6 h in the presence of anti-CD28 and CD49d mAbs as well as 5-hour GolgiPlug treatment as described above [20]. Cultured cells were then subject to surface and intracellular staining.

To ensure the specific immune staining in direct or conventional ICS, matched normal serum or isotype IgG served as negative controls for staining cytokines or surface markers.

### Determination of bacterial CFU counts from lung tissues

To measure bacilli counts in lung tissues, a half of cut-sections of the right caudal, right middle, or the left caudal lobes from each animal were taken for CFU determination after the extensive gross pathologic evaluation was accomplished. If there were tuberculosis lesions in the respective lobe, a half of the lung tissue containing approximately 50% lesions was taken. If no visible lesions were seen in the respective lobe, a random half of tissue was taken for evaluation. Tissue homogenates were made diluted and plated as described previously [10]. The plates were incubated in a 37°C incubator for 3 weeks, and CFU was counted.

### Gross pathologic analyses of TB lesions, scoring systems, and histopathological analysis

The approaches and procedures were essentially the same as we previously described [8,21]. Briefly, animals were euthanized by intravenous barbiturate overdose, and immediately necropsied in a biological safety cabinet. Standard gross pathologic evaluation procedures were performed by the blinded medical pathologists/associates (LS, DH), with each step recorded and photographed. Lung lobes, bronchial, mesenteric, axillary and inguinal lymph nodes, tonsils, and other major organs were collected and labelled. Multiple specimens from all tissues with gross lesions and remaining major organs were harvested. Gross observations including but not limited to the presence, location, size, number, and distribution of lesions were recorded.

The scoring system was excised to calculate gross pathology scores for TB lesions as previously described [7,21].

The unbiased processes for collecting/preparing tissues for histopathology sections, HE staining procedures, and images were performed as we previously described [7,22].

### Statistical analysis

Statistical analysis was done by using GraphPad Prism software (GraphPad Software, Inc., La Jolla, CA). Data were analyzed using Mann–Whitney test (non-parametric method or ANOVA, as we previously described) [7,22]. *p* < 0.05 was considered significant. Only *p*-values <0.05 were shown in the text.

### Study approval

The use of animals and all experimental procedures were approved by Institutional Animal Care and Use Committee and Biosafety Committees at Wuhan University [10].

## Results

### ZOL/IL-2 treatment remarkably activated and expanded Vγ2Vδ2 T cells in PBL of Chinese cynomolgus macaques, and enhanced effector capabilities of Vγ2Vδ2 T cells to produce anti-TB cytokines and to inhibit intracellular mycobacterial growth

**Figure 2.**
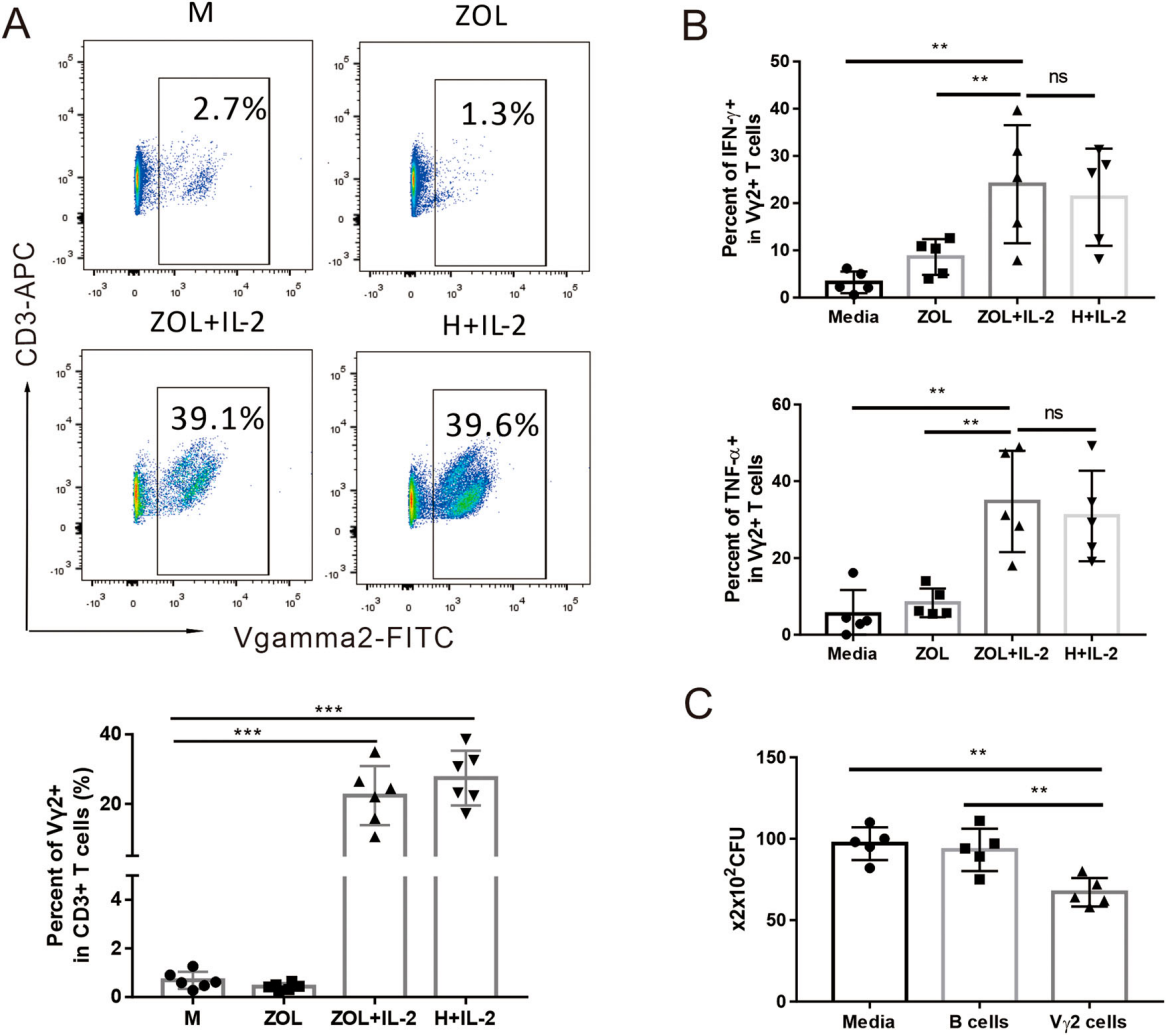
ZOL/IL-2 treatment remarkably activated and expanded Vγ2Vδ2 T cells in PBL of Chinese cynomolgus macaques, and enhanced effector capabilities of Vγ2Vδ2 T cells to produce cytokines and to inhibit intracellular mycobacterial growth. (A) Representative flow cytometry histograms show that ZOL, as well as phosphoantigen (HMBPP), could activate and expand Vγ2Vδ2 T cells in PBMC from Chinese cynomolgus macaques in 7-day co-culture with IL-2. Data were gated on CD3. (B) ZOL/IL-2-expanded Vγ2Vδ2 T effector cells could produce appreciable levels of anti-TB cytokines IFN-γ and TNF-α. (C) ZOL/IL-2-expanded Vγ2Vδ2 T cells could inhibit intracellular mycobacterial (BCG) growth. Data are shown as means ± SEM in three independent experiments. ** *p* < 0.01, * *p* < 0.05.

To examine whether zoledronate plus IL-2 (ZOL/IL-2) could expand Vγ2Vδ2 T cells of Chinese cynomolgus macaques, PBMC cells from macaques blood were treated with ZOL plus IL-2 for 7 days as we previously described [16,20]. Results from flow cytometry analysis showed that the percentages of Vγ2Vδ2 T cells in CD3 T cells expanded to ∼20–30% in ZOL/IL-2 treated PBMC, and were similar to those treated by microbial phosphoantigen HMBPP (H) plus IL-2 (Figure 2(A)). As described in our multiple publications, phosphoantigen-expanded Vγ2 or Vδ2 T cells mostly co-expressed Vγ2Vδ2 TCR heterodimers. This also applied to ZOL-expanded Vγ2 T subset, which was thus interpreted/described as Vγ2Vδ2 T cells here in this manuscript. Note that medium (M) or ZOL alone control, like IL-2 alone control in our earlier publications, did not remarkably expand Vγ2Vδ2 T cells.

Moreover, IFN-γ-producing Vγ2Vδ2 T effector cells were increased to more than 20%, which were about 10 times in unstimulated Vγ2Vδ2 T cells (Figure 2(B)). Similarly, ZOL/IL-2 expanded much higher percentages of TNF-α-producing Vγ2Vδ2 T effector cells than control (Figure 2(B)).

We then sought to assess ZOL/IL-2-expanded Vγ2Vδ2 T cells for anti-Mtb effector function. To this end, we determined whether these Vγ2Vδ2 T cells exhibited an increased ability to mount fast-acting immunity against TB infection using a cellular model [23]. For proof-of-concept, we transiently co-cultured both the isolated Vγ2Vδ2 T cells and the BCG-infected A549 cells as a fast-acting innate immunity model and tested the ability of ZOL/IL-2-expanded Vγ2Vδ2 T cells to limit/control intracellular mycobacterial growth in infected A549 lung cells as we previously described [23]. Use of BCG-infected lung cells in multiple experiments would help to reduce variations of both bacilli uptake and monocytes numbers from different donor’s PBMC compared to direct *Mtb* exposure to PBMC under BSL-III condition. Thus, ZOL/IL-2-expanded Vγ2Vδ2 T cells were isolated, then co-cultured for 3 days with BCG-infected A549 lung-epithelial cells, and then assessed for CFU counts in lysate of co-cultured cells. As expected, BCG CFU counts in co-cultures from ZOL/IL-2-expanded Vγ2Vδ2 T cells were significantly lower than control groups (Figure 2(C)).

The results above implicated that ZOL/IL-2 treatment in PBMC culture remarkably expanded macaque Vγ2Vδ2 T cells, and enhanced effector capabilities of Vγ2Vδ2 T cells to produce anti-TB cytokines and to inhibit intracellular mycobacterial growth, although this effect was not formally tested with Mtb.

### Adjunctive ZOL/IL-2 administrations during TB drugs treatment of MDR-TB-infected macaques could significantly increase circulating Vγ2Vδ2 T cells from week 2 through week 14

Our previous proof-of-concept studies demonstrated that phosphoantigen expansion and differentiation of Vγ2Vδ2 T-cell subset could increase resistance to tuberculosis [7]. To examine the utility for γδ T-cell-targeted therapeutics against refractory MDR-TB, each of the studied macaques was infected with 500 CFU of clinical MDR-TB strain V791 as we previously described [10], and were then treated with ZOL/IL-2 (clinical drugs for human diseases) as adjunctive immunotherapy in combination with second-line anti-TB drugs against MDR-TB (Table 1 and Supplemental Table). As the test group(Group-2), ZOL/IL-2 administrations plus anti-TB drugs were comparatively evaluated with control groups, namely anti-TB drugs alone (Group 1), IL-2 alone plus anti-TB drugs (Group 3) and saline only (Group 4) (Table 1). Clinical practice and publications [12] indicate that fresh or primary TB is more readily controlled by multiple sensitive anti-TB drugs (antibiotics) than MDR-TB in patients, and that TB antibiotics can directly kill/suppress TB or MDR-TB bacilli inside and outside target cells and therefore more rapidly/effectively control TB infection than immunotherapy drug alone because the later indirectly acts on T cells with no or less killing of TB or MDR-TB bacilli. To avoid the ability of TB drugs to overshadow potential immunotherapeutic effects [8,12], we adopted intermittent treatments with TB drugs, with short-term intervals (without TB drugs) in 12-day treatment cycles (Table 1 and Supplemental Table). Such short-term omission of TB drugs before the next treatment cycle presumably would result in a low or residual level of MDR-TB infection, and such a setting would make it more readily to determine whether adjunctive ZOL/IL-2 or IL-2 regimen could exert better therapeutic effects than TB drugs alone.

**Figure 3.**
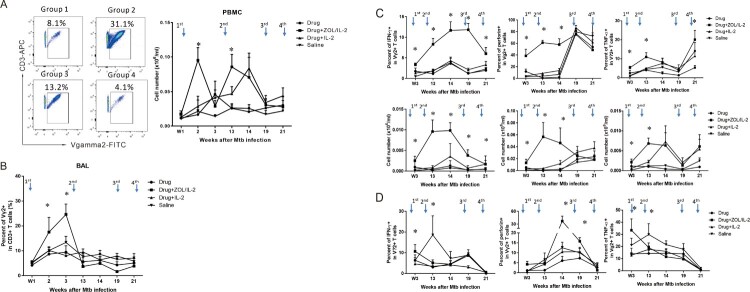
Adjunctive ZOL/IL-2 administrations significantly expanded Vγ2Vδ2 T cells from week 2 through week 14 after infection, and enhanced/sustained their effector functions of producing anti-TB cytokines until week 21, with some Vγ2Vδ2 T effector subpopulations detected in airway at weeks 3, 14, 19. (A). On the left are representative flow cytometry histograms showing that ZOL/IL-2 administration in TB drug-treated macaques (Group-2) induced greater expansion of Vγ2Vδ2 T cells (% of CD3+) in PBMC at week 2 compared to controls (Group-1, −3, −4). On the right are the graph data showing mean absolute numbers of Vγ2Vδ2 T cells in PBMC over time after MDR-Mtb strain V791 infection and treatments with ZOL/IL-2 (Group-2) or controls (Group-1, −3, −4). Note that the first and second ZOL/IL-2 administrations significantly increased circulating Vγ2Vδ2 T cells at weeks 2 and 13, and that even at week 14, ZOL/IL-2-treated Group-2 still showed significantly higher numbers of Vγ2Vδ2 T cells than controls Group-1 and Group-4. Arrows in the top indicate the time points for administering ZOL/IL2 or control items, whereas intermittent treatments with TB drugs were described in the text. (B). Graph data show that mean percentages of airway Vγ2Vδ2 T cells in BAL fluid from Group 2 were significantly higher than those in other groups at the early stage (initial 3 weeks) after MDR-Mtb V791 infection. (C). Graph data show that adjunctive administration of ZOL/IL-2 in Group-2 macaques could significantly increase and sustain mean percentages (upper panels) and absolute numbers (lower panels) of circulating Vγ2Vδ2 T effector cells capable of producing anti-TB cytokine IFN-γ (left) from week 3 until week 21 after MDR-Mtb V791 infection, when compared to controls groups. Note that Vγ2Vδ2 T effector cells producing anti-TB perforin and TNF-α were also significantly increased in PBMC of Group-2 until weeks 14 and 21, respectively. (D). Graph data show that adjunctive administration of ZOL/IL-2 in Group-2 macaques could inconsistently increase numbers of airway Vγ2Vδ2 T effector cells capable of producing IFN-γ, perforin or TNF-α at weeks 3, 14, or 19 in BAL fluid after MDR-Mtb V791 infection, when compared to controls groups.

To determine whether ZOL/IL-2 regimen could activate and expand Vγ2Vδ2 T cells in high-dose MDR-TB infection, the 1st administration (Table 1) began at week 1 (day 7) after infection, and was assessed for increases in percentages and absolute numbers of Vγ2Vδ2 T cells. We found that ZOL/IL-2 administration in Group-2 macaques after MDR-TB infection/TB drug treatment induced significantly greater percentage and absolute numbers of Vγ2Vδ2 T cells in PBMC until week 14, when compared to controls (Figure 3(A)). In fact, the first and second ZOL/IL-2 administrations led to significantly greater increases in circulating Vγ2Vδ2 T cells than TB drugs alone, IL-2 alone and saline controls at weeks 2 and 13 (Figure 3(A)). Even at week 14, ZOL/IL-2-treated Group-2 still showed significantly greater numbers of Vγ2Vδ2 T cells than controls Group-1 and Group-4 (Figure 3(A)). While the third and fourth treatments only led to subtle increases compared to baseline levels and the saline control (Figure 3(A)), such subtle increases appeared to correspond to the significantly-greater numbers of IFN-γ-producing Vγ2Vδ2 T effector cells in the test Group-2 (Figure 3(C), left).

To examine whether ZOL/IL2 administration also increased Vγ2Vδ2 T cells in the pulmonary compartment, we measured percentage numbers of Vγ2Vδ2 T cells in BAL fluid collected at different time points. Results showed that after the first-dose ZOL/IL-2 administration, the mean percentage of Vγ2Vδ2 T cells in BAL of Group-2 macaques was the highest among four groups at week 3, the time the expanded Vγ2Vδ2 T cells in PBMC started undergoing dynamic changes for T-cell homeostasis (Figure 3(B)). Although Vγ2Vδ2 T cells in BAL fluid remained low in subsequent ZOL/IL-2 expansions of circulating γδ T cells in PBMC, we could not exclude the possibility that activated Vγ2Vδ2 T effector cells (Figures 3(C,D)) might accumulate in lung mucosae instead of airway (BAL fluid) as detected in our previous studies [6,24]. These results suggest that the administration of ZOL/IL-2 could induce the *in vivo* expansion of Vγ2Vδ2 T cells despite MDR-Mtb V791 infection, and that the expanded Vγ2Vδ2 T cells could traffic to airway (the BAL fluid), the part of the pulmonary compartment.

### Adjunctive ZOL/IL-2 administrations during intermittent TB drugs treatment of MDR-TB-infected macaques could significantly enhance and sustain Vγ2Vδ2 T-effector subpopulations producing anti-TB cytokines from week 3 through week 21

It has been reported that Vγ2Vδ2 T cells play an important role in anti-TB immunity through producing anti-TB effector cytokines and CTL killing [25]. To examine whether *in vivo* ZOL/IL-2-expanded Vγ2Vδ2 T cells could produce anti-TB cytokines, we employed the modified direct ICS assay without *in vitro* antigen stimulation so that the detected T effector cells were similar to the *in vivo* setting during infections [8,21].

Adjunctive administration of ZOL/IL-2 in Group-2 macaques could significantly increase and sustain mean percentages and absolute numbers of circulating Vγ2Vδ2 T effector cells capable of producing anti-TB cytokine IFN-γ from week 3 until week 21 after MDR-Mtb V791 infection, when compared to controls groups (Figure 3(C), left panels). In addition, Vγ2Vδ2 T effector cells producing anti-TB perforin and TNF-α were also significantly increased in PBMC of Group-2 until weeks 14 and 21, respectively (Figure 3(C), mid and right panels). Furthermore, adjunctive administration of ZOL/IL-2 in Group-2 macaques could increase numbers of airway Vγ2Vδ2 T effector cells capable of producing IFN-γ, perforin, and TNF-α at week 3, with perforin-producing Vγ2Vδ2 T effector population consistently increased in airway at weeks 3, 14, 19, when compared to controls groups (Figure 3(D)). The less-consistent changes in Vγ2Vδ2 T effectors in BAL fluid during adjunctive ZOL/IL-2 administrations might be explained by the activation-induced T-cell homeostasis or exhaustion. Alternatively, this would be explained by the possibility that Vγ2Vδ2 T effector cells might accumulate in lung mucosae as detected in our previous study [24] rather than in airway (BAL fluid), because the IFN-γ-producing Vγ2Vδ2 T effector cells were detected at higher levels during the entire ZOL/IL-2 intervention of MDR-TB infection (Figure 3(D), left panels). Together, the results suggest that adjunctive ZOL/IL2 administrations could significantly enhance and sustain enhance and sustain the Vγ2Vδ2 T-effector population producing anti-TB cytokines from week 3 through week 21, with some Vγ2Vδ2 T effector subpopulations trafficking to airway at weeks 3, 14, 19.

### Adjunctive ZOL/IL-2 administrations, while expanding Vγ2Vδ2 T cells, significantly increased/sustained numbers of circulating CD4^+^Th1 and CD8^+^Th1-like effector populations, with some αβ T-effector populations detected in airway at weeks 3 and 14

Since activated Vγ2Vδ2 T cells could function as antigen presenting cells (APC) to present peptide antigens to αβ^+^ T cells [26], we wanted to know whether ZOL/IL2 activation of Vγ2Vδ2 T cells could lead to enhanced immune responses of αβ+ T cells against MDR-Mtb infection. To this end, we measured changes in numbers of cytokine-producing CD4^+^ and CD8^+^ T effector cells using the direct ICS methods without antigen stimulation in culture.

**Figure 4.**
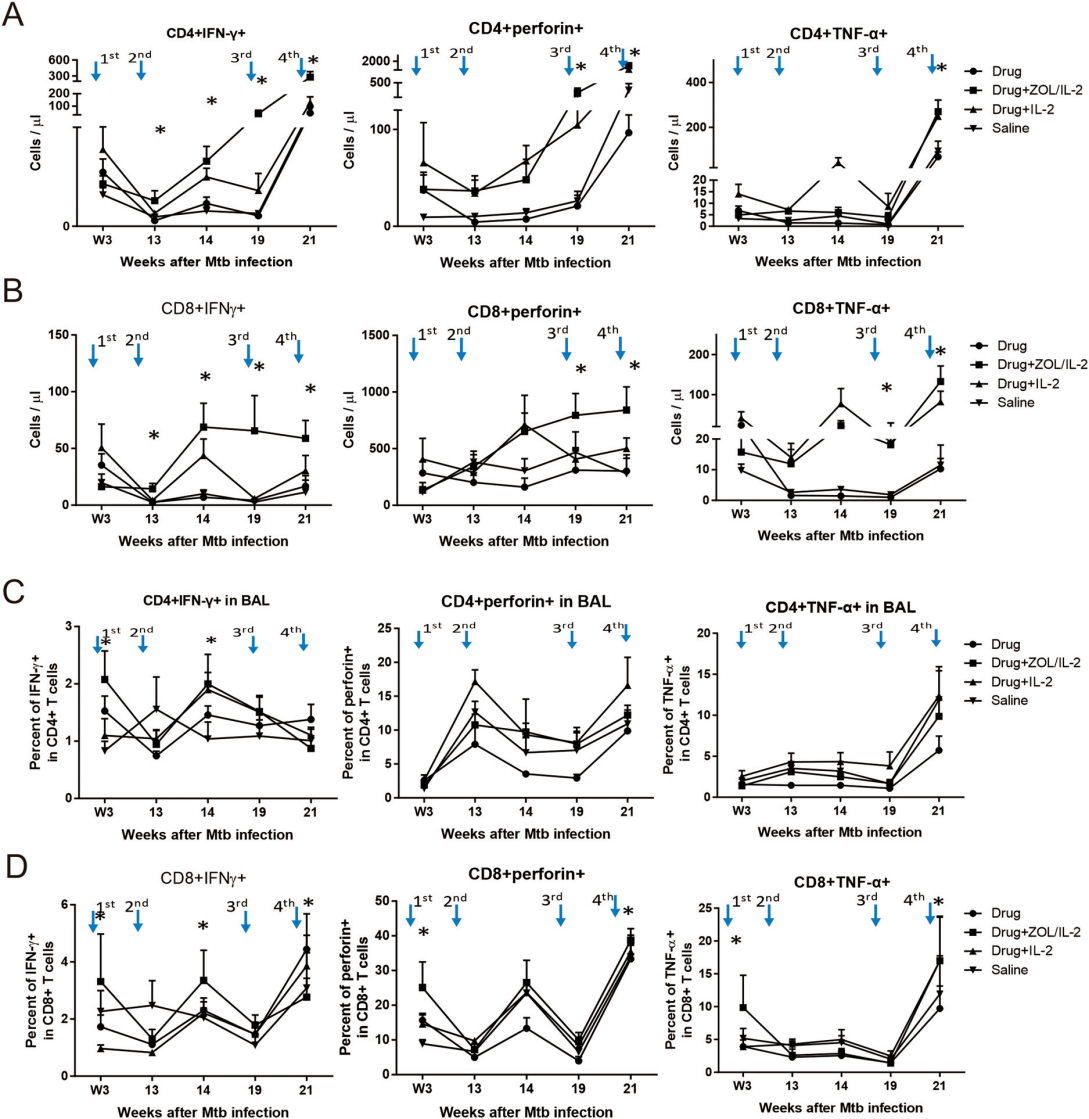
Administration of ZOL/IL-2, while specifically expanding Vγ2Vδ2 T cells, could significantly increase/sustain mean numbers of CD4^+^ Th1 and CD8^+^ Th1-like effector cells in PBMC until week 21, with some αβ T-effector populations detected in airway at weeks 3 and 14. (A)–(B). Graph data show that administration of ZOL/IL-2 in Group-2 macaques after MDR-Mtb V791 infection significantly increased and sustained mean absolute numbers of IFN-γ-producing CD4^+^ Th1 cells (A, left) and CD8^+^ Th1-like cells (B, left) in PBMC from week 13 until endpoint week 21 after MDR-Mtb V791 infection, when compared to controls groups (except for Group-3 at week 14). ZOL/IL-2 administration also significantly increased CD8^+^ T effector cells producing perforin (B, middle) and TNF-α (B, right) at late-phase weeks 14 and 21, with inconsistent increases in CD4^+^ T cells producing perforin (A, middle) or TNF-α (A, right) in one of these two time points. (C)–(D). Graph data show that administration of ZOL/IL-2 in Group-2 macaques after MDR-Mtb V791 infection significantly increased mean absolute numbers of IFN-γ-producing CD4^+^ Th1 cells (C, left) and CD8^+^ Th1-like cells (D, left) in BAL fluid at weeks 3 and 14 after MDR-Mtb V791 infection, when compared to saline control Group-4.

Interestingly, adjunct administration of ZOL/IL2 in Group-2 macaques after MDR-Mtb V791 infection significantly increased and sustained absolute numbers of IFNγ-producing CD4^+^ Th1 cells (Figure 4(A), left) and CD8^+^ Th1-like cells (Figure 4(B), left) in PBMC from week 13 until week 21 after MDR-Mtb V791 infection, when compared to controls groups (except for Group-3 at week 14). ZOL/IL2 administration also significantly increased CD8^+^ T effector cells producing perforin (Figure 4(B), middle) and TNF-α (Figure 4(B), right) at late-phase weeks 14 and 21, with inconsistent increases in CD4^+^ T cells producing perforin (Figure 4(A), middle) or TNF-α (Figure 4(A), right) in one of these two-time points.

Notably, adjunct administrations of ZOL/IL2 in Group-2 macaques promoted an earlier increase in numbers of airway CD4^+^ Th1 cells and CD8^+^ Th1-like effector cells capable of producing the anti-TB cytokine IFN-γ in BAL fluid at weeks 3 after infection, and sustained the increase in them at week 14 thereafter, when compared to saline control Group-4 (left panels in both Figure 4(C,D)). It is also noteworthy that ZOL/IL2 regimen enhanced earlier increases in airway CD8^+^ T effector cells producing both perforin and TNFα (Figure 4(D), mid and right panels).

Thus, the above results obtained by direct ICS without *in vitro* antigen stimulation suggest that ZOL/IL2 activation/expansion of Vγ2Vδ2 T cells led to the enhanced ability of αβ CD4^+^ and CD8^+^ T cells to constitutively produce anti-TB cytokines and that some αβ T-effector populations trafficked to airway at weeks 3 and 14 after MDR-TB infection.

### Adjunctive ZOL/IL2 administration antagonized the IL-2’s capability to early expand CD4^+^ CD25^+^ Foxp3^+^ Treg cells, but subsequently permitted increases in IL2-induced Treg or Foxp3^+^ T cells, with consistent increases in CD25^+^ T effector cells

IL-2 has been shown to expand T effector cells for anti-microbial/anti-cancer immunity, while IL-2 can also induce or grow CD4^+^ CD25^+^ Foxp3^+^ regulatory T cells for immune regulation or tolerance [27,28]. While *in vitro* ZOL treatment could reduce numbers of Treg cells and Treg suppression in cultured PBL from cancer patients [29], it remains unknown whether *in vivo* ZOL or ZOL/IL-2 administration can regulate Treg cells. To address this, we comparatively examine whether IL-2 and ZOL/IL-2 administrations could induce and regulate CD4^+^ CD25^+^ Foxp3^+^ Treg cells, CD8^+^ CD25^+^ Foxp3^+^ or Vγ2^+^ CD25^+^ Foxp3^+^ T cells.

**Figure 5.**
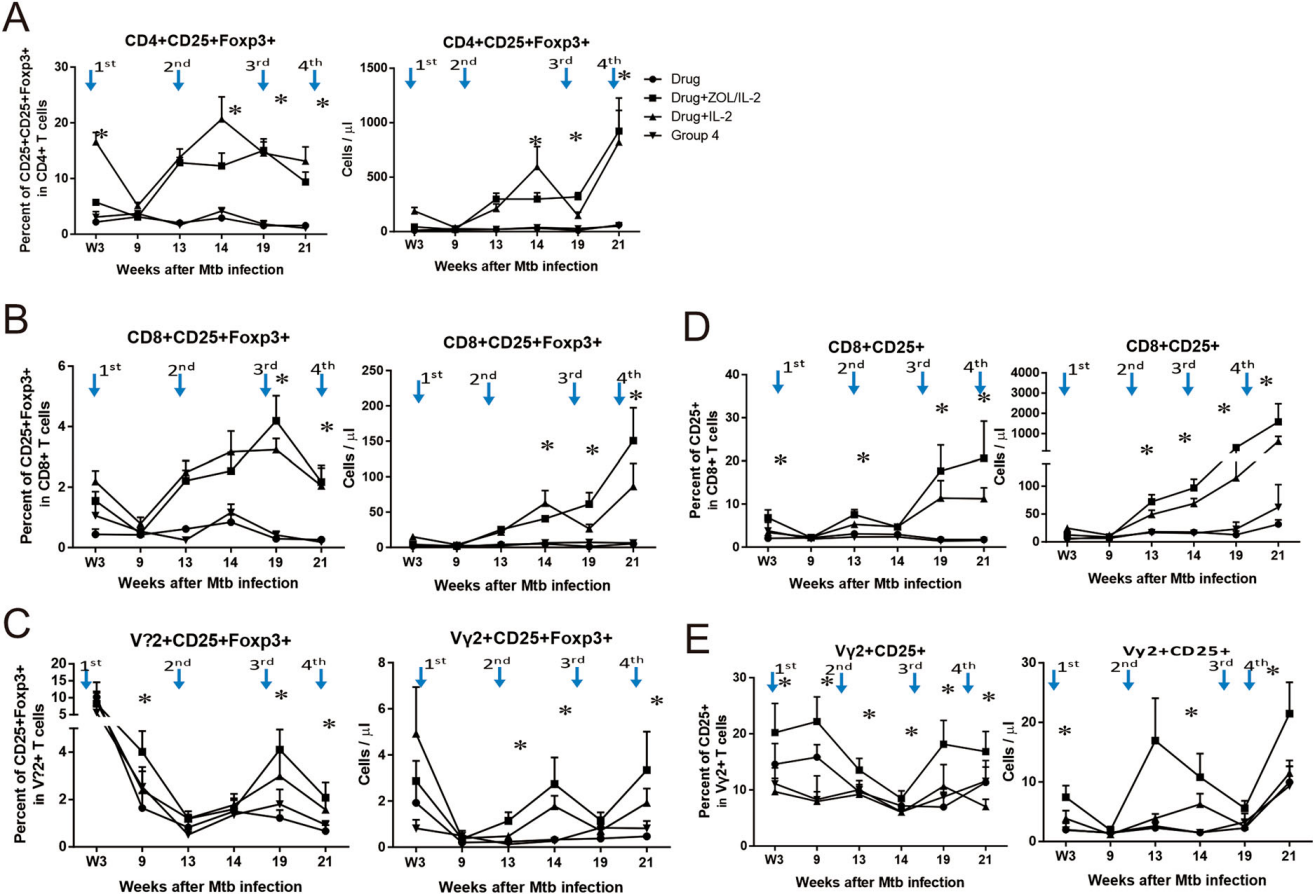
Adjunctive ZOL/IL-2 administrations antagonized the IL-2’s capability to early expand CD4^+^ CD25^+^ Foxp3^+^ Treg cells, but subsequently permitted increases in IL-2-induced Treg or Foxp3^+^ T cells, with consistent increases in CD25^+^ T effector cells. (A). Shown were percentage (left graph) and absolute (right) numbers of circulating CD4^+^ CD25^+^ Foxp3^+^ Treg cells. ZOL/IL-2 administration after MDR-Mtb infection in Group-2 macaques remarkably reduced the ability of IL-2 to expand CD25^+^ Foxp3^+^ Treg cells at week 3 compared to Group-3, and subsequently permitted increases in IL-2-induced Treg cells at weeks 13, 14, 19, 21, when compared to drug-alone and saline controls. Note that ZOL/IL-2 group showed significantly lower numbers of Treg cells than IL-2 alone group at week 3 (*, *p* < 0.05 for comparison between Groups 2 and 3), and significantly upheld increased numbers of IL-2-induced Tregs from week 13 through week 21 (*, *p* > 0.05 for comparisons between Group-2 and Group-1 or Group-4; however, *p* > 0.05 for comparisons between Groups-2 and -3 except at week 14). (B). Graph data of percentage (left) and absolute (right) numbers show that ZOL/IL-2 (Group-2) or IL-2 alone (Group-3) administrations induced significant increases in circulating CD8^+^ CD25^+^ Foxp3^+^ T cells at weeks 14, 19, and 21 after MDR-Mtb infection, compared to Group-1 or Group-4 (*, *p* < 0.05). (C). Graph data of percentage (left) or absolute (right) numbers show that ZOL/IL-2 (Group-2) or IL-2 alone (Group-3) administrations induced significant increases in circulating Vγ2^+^ CD25^+^ Foxp3^+^ T cells at weeks 13, 14, 19 and 21 after MDR-Mtb infection, compared to Group-1 or Group-4 (*, *p* < 0.05). (D). Graph data of percentage (left) or absolute (right) numbers show that ZOL/IL-2 (Group-2) or IL-2 alone (Group-3) administrations induced significant increases in circulating CD8^+^ CD25^+^ T effector cells at weeks 3, 13, 14, 19, and 21 after MDR-Mtb infection, compared to Group-1 or Group-4 (*, *p* < 0.05). (E). Graph data of percentage (left) or absolute (right) numbers show that ZOL/IL-2 (Group-2) administrations induced significant increases in circulating Vγ2^+^ CD25^+^ T effector cells at weeks 3, 9, 13, 14, 19, and 21 after MDR-Mtb infection, compared to Group-1, Group-3 or Group-4 (*, *p* < 0.05).

While IL-2 administration in Group-3 macaques could induce or expand CD4^+^ CD25^+^ Foxp3^+^ Treg cells as we previously described [8], ZOL/IL-2 administrations after MDR-TB infection in Group-2 macaques reduced the ability of IL-2 to expand CD25^+^ Foxp3^+^ Treg cells at week 3 (Figure 5(A)). Interestingly, ZOL/IL-2 administrations in Group-2 macaques subsequently permitted increases in IL2-induced Treg cells at weeks 13, 14, 19, 21, when compared to drug-alone and saline controls (Figure 5(A)). In fact, this ZOL/IL-2-treated group significantly upheld increased numbers of IL-2-induced Tregs from week 13 through week 21 (*p* < 0.05 for comparisons between Group-2 and Group-1 or Group-4; however, *p* > 0.05 for comparisons between Groups-2 and -3 except at week 14). The bi-phasic regulations of Foxp3^+^ Treg by ZOL + IL2 administrations may be driven by magnitudes of Vγ2Vδ2 T-cell expansion (Figure 3(A), Figure 3(B), Figure 5(A)). In addition, administrations of ZOL/IL-2 (Group-2) or IL-2 (Group-3) after MDR-TB infection induced significant increases in circulating CD8^+^ CD25^+^ Foxp3^+^ T cells at weeks 14, 19, and 21, compared to Group-1 or Group-4 (*p* < 0.05, Figure 5(B)). Concurrently, ZOL/IL-2- or IL-2-treated groups exhibited significant increases in circulating Vγ2^+^ CD25^+^ Foxp3^+^ T cells at weeks 13, 14, 19, and 21, compared to Group-1 or Group-4 (*p* < 0.05, Figure 5(C)).

It has been reported that activated T effector cells expressing CD25, the α-chain of high affinity IL-2 receptor, gain effector function when lacking the expression of the Treg marker Foxp3 [30]. We therefore examined whether ZOL/IL-2 or IL-2 regimen could increase the frequencies of CD25^+^ T effector cells. In fact, adjunctive administrations of ZOL/IL-2 in Group-2 or IL-2 in Group-3 induced significant increases in circulating CD8^+^ CD25^+^ T effector cells at weeks 3, 13, 14, 19, and 21, compared to Group-1 or Group-4 (*, *p* < 0.05, Figure 5(D)). Consistently, ZOL/IL-2 administration in Group-2 induced significant increases in circulating Vγ2^+^ CD25^+^ T effector cells at weeks 3, 9, 13, 14, 19, and 21, compared to Group-1, Group-3, or Group-4 (*, *p* < 0.05, Figure 5(E)).

Together, the results above implicate that adjunctive ZOL/IL-2 administration antagonized the IL-2’s capability to early expand CD25 + Foxp3 + Treg cells at week 3, but subsequently permitted increases in IL2-induced Treg or Foxp3 + T cells, with consistent increases in CD25 + T effector cells.

### Adjunctive ZOL/IL-2 administrations after MDR-TB infection led to lower MDR-TB bacterial burdens in lungs than TB drugs alone, IL-2 alone, or saline controls, and resulted in body weight gains at weeks 17–21

We then sought to determine whether adjunctive ZOL/IL-2 administrations, while expanding anti-TB Vγ2Vδ2 T effector cells and facilitating immune responses of CD4^+^ Th1 or CD8^+^ T effectors, led to immune resistance to MDR-Mtb V791 infection. We clinically followed all four groups of macaques during the MDR-TB infection, and conducted complete necropsy studies as we previously described [7,22], starting at month 5 (149th day) after infection.

To optimally uncover potential differences MDR-Mtb V791 infection and TB lesions between adjunctive ZOL/IL-2 or IL-2 and controls, each macaques in four studied groups were injected subcutaneously with Adalimumab during the last 4 weeks before the endpoint [13]. Such unbiased Adalimumab treatment allowed for enhancing and activating any inactive or subclinical MDR-TB infection that had been potentially contained through ∼4 months of intermittent TB drug treatments and adjunctive immune interventions.

**Figure 6.**
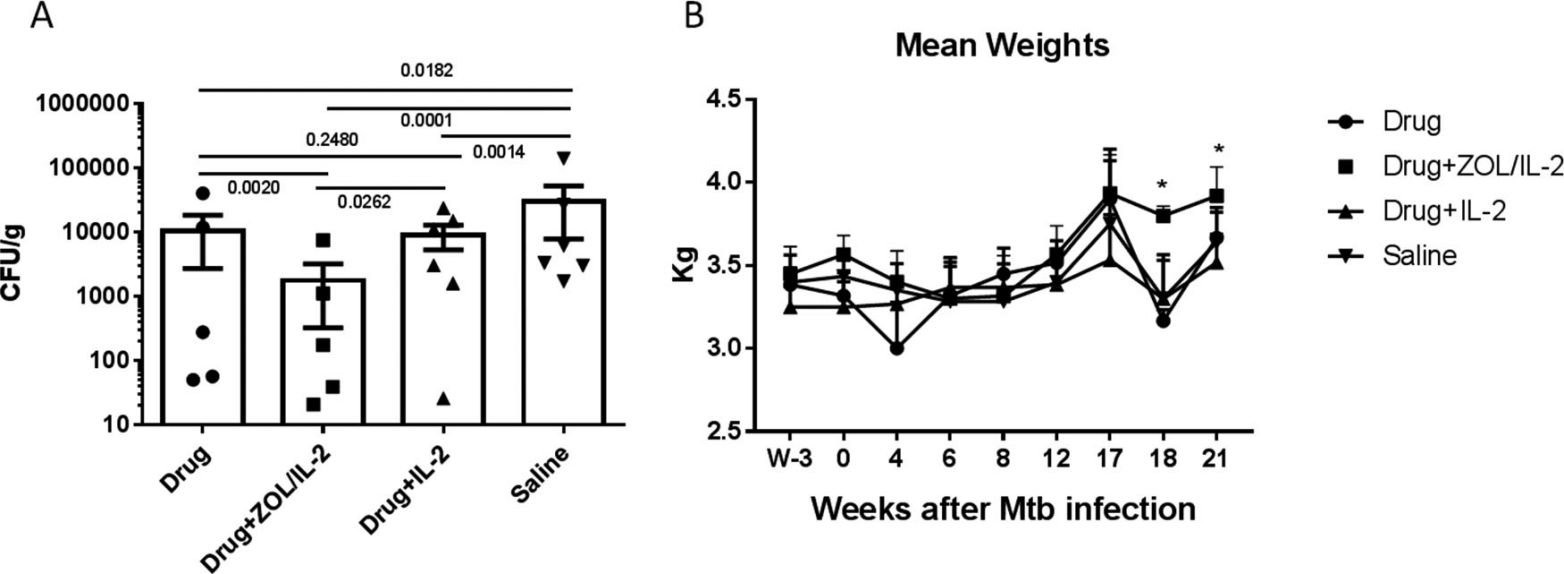
Adjunctive ZOL/IL-2 administrations after MDR-Mtb infection led to lower MDR-Mtb bacterial burdens in lungs than TB drugs alone, IL-2 alone or saline controls, and resulted in body weight gains at weeks 17-21. (A). Graph data show mean CFU counts ± SEM per gram of lung tissue homogenates for right lung lobes obtained at endpoints from 4 groups of MDR-Mtb-infected macaques. To optimally uncover potential differences between groups in MDR-Mtb V791 infection and TB lesions, each of macaques in four studied groups were injected subcutaneously with Adalimumab during the last 4 weeks before the endpoint [13]. Such unbiased Adalimumab treatment allowed for enhancing and reactivating any inactive or latent MDR-Mtb infection that had been potentially contained through ∼4 months of intermittent TB drug treatments and immune-based interventions. Statistical analysis was done using ANOVA test, with *p*-values indicated for comparisons between groups. (B). The graph data show changes in mean body weights for 4 groups of macaques over time after MDR-Mtb strain infection and treatments. Baseline values for body weights were shown as 0, with positive changes indicating weight gains relative to the baseline body weights, and negative ones being weight loss. Based on published TB studies in cynomolgus macaques, consecutive changes in >0.25 (>25%) were considered significant.

At the endpoint, we employed the peer-advocated efficacy evaluation strategy/principle for necropsy, tissue collections, CFU measurements, and pathology evaluation as we previously described [7,13,22]. Thus, we measured MDR-Mtb bacterial CFU counts per gram of lung tissue homogenates in right lung lobes obtained at endpoints from 4 groups of MDR-Mtb-infected macaques. Our results demonstrated that ZOL/IL-2-treated macaques (Group-2) exhibited significantly lower mean CFU counts in lungs than TB drugs only (Group-1), IL-2 plus TB drugs (Group-3) or saline (Group-4) control (*p* < 0.05 by ANOVA test, Figure 6(A)). The lower MDR-TB infection level in ZOL/IL-2-treated Group-2 macaques was consistent with the clinical follow-up status. In fact, the ZOL/IL-2-treated group exhibited a sustained gain of body weights at weeks 17–21 after MDR-TB V791 infection (Figure 6(B)).

Thus, these results suggest that adjunctive ZOL/IL-2 therapy could enhance immune responses of anti-TB Vγ2Vδ2 T, CD4 + Th1 and CD8 + T effector populations, and led to lower MDR-TB bacterial burdens with favourable clinical outcome as body weight gains.

### Adjunctive ZOL/IL-2 (Group-2) or IL-2 (Group-3) administrations after MDR-TB infection and TB drug treatment led to milder MDR-TB pathology/lesions compared to TB drug alone and saline controls

Our results above already demonstrated that adjunctive ZOL/IL-2 regimen could enhance immune responses of anti-TB Vγ2Vδ2 T, CD4 + Th1 and CD8 + T effector populations as well as Foxp3+ Tregs. We also showed that ZOL/IL-2 regimen attenuated MDR-TB infection with favourable clinical outcome. Here, we sought to determine if ZOL/IL-2-enhaced responses of Vγ2Vδ2 T effectors, CD4 + Th1, CD8 + T effectors and Foxp3+ Tregs could lead to milder MDR-TB pathology or lesions. We therefore conducted comparative pathology studies of four macaque groups following the procedures/strategies described previously [7,22]. Whole-lung images showed that all macaques in saline control group exhibited severe TB tubercles (large black arrows) or diffuse haemorrhages together with TB tubercules (red arrows) (Figure 7(A)). The cut-sections (Figure 7(B)) in the right caudal lung (infection site) confirmed both the severe MDR-TB lesions as caseation miliary TB or extensive coalescing TB tubercules (black arrows), and the diffuse haemorrhage with coalescing TB granulomas (red arrows). Notably, three macaques in the control Group-1 (TB drugs alone) exhibited mid-size TB tubercles (white arrows) and diffuse/focal haemorrhages (large/small pink arrows) (Figure 7(A,B)). In contrast, ZOL/IL-2-treated (Group-2) and IL-2-treated (Group-3) did not develop any gross haemorrhages or caseation miliary TB or extensive coalescing TB tubercules (Figure 7(A,B)). Only 2 macaques in Group-2 or Group-3 showed mid-side (white arrow) or small (small black arrows) TB granulomas (Figure 7(A,B)). It is noteworthy that massive or mild haemoptysis is well described as TB-associated haemorrhages in some TB patients [31]. Here, the haemorrhages in control Group-1 (TB drugs only) and Group-4 (saline) appeared to be induced or promoted by the endpoint Adalimumab treatment, because Adalimumab allowed for enhancing MDR-TB lesions or activating subclinical/inactive TB infection as described in humans and nonhuman primates [13]. This notion helps to explain why Group-2 (ZOL/IL-2) and Group-3 (IL-2) macaques who exhibited milder TB pathology did not develop haemorrhages after Adalimumab treatment. Figure 7.Adjunctive ZOL/IL-2 (Group-2) or IL-2 (Group-3) administration after MDR-Mtb infection and TB drug treatment led to milder MDR-TB pathology/lesions compared to TB drugs alone and saline controls. (A) Shown are representative digital images of whole lungs obtained at end time points for individual MDR-TB-macaques in 4 studied groups, with animal ID displayed in the upper-left corner. The vertical/horizontal bars at the bottom left represent the 1-cm scale derived from the fluorescence rulers of each original photo, including the sliced sections. Large red arrows indicate diffuse haemorrhages with tubercle lesions; white arrows denote gross tubercle lesions; pink arrows show mid-size haemorrhage changes; small black arrows point to focal gross tubercle lesions. All macaques in saline group exhibited typical severe TB tubercles as shown, whereas representative changes were displayed for other groups. (B). Representative digital images of the cut-sections of the right caudal lung lobes from 3 representative MDR-Mtb V791-infected macaques in each of four groups, with the ID numbers indicated in the upper-left corner. Note that the lung lobes of MDR-Mtb-infected macaques were sliced into exhibition sections, because more stringent safety protocols had to be adhered in the setting of MDR-Mtb infection. TB lesions could be adjudged based on the examples indicated by the various colour arrows: large black arrows demonstrated the presence of caseation TB pneumonia or extensive coalescing TB granulomas; large red arrows point to the diffuse haemorrhage with coalescing TB tubercules/lesions; large pink arrows indicate diffuse haemorrhage changes without coalescing tubercles; white arrows indicate coalescing TB tubercles; small pink arrows denote focal haemorrhages; small black arrows point to the small noncoalescing TB granulomas lesions. (C). Graph data of mean gross pathology scores ± SEM for 4 groups of MDR-Mtb-infected macaques (*n* = 6). The scores were calculated as we previously described [7,22]. Statistical analysis was done using ANOVA test, with *p* values indicated for comparisons between groups. (D)–(E). Representative histopathological images of H&E-stained lung sections in the right caudal lobes collected from representative MDR-Mtb-infected macaques in 4 studied groups. The upper and lower panels showed 50× (D) and 200× (E) magnifications, respectively, for different right caudal lung lobe sections of 3 representative macaques in each of 4 studied groups. Overall, the sections from the saline group of macaques exhibited severe TB lesions (large black arrows) and different stages of haemorrhagic changes (large red arrows). The recent haemorrhages displayed residual erythrocytes/debris or pigment haemoglobin; earlier haemorrhage appeared to be predominated by post-haemorrhage necrosis or destruction. The severe TB lesions (such as WPA20, WPA21) were characterized by widespread necrosis and tissue destruction, with many epithelioid cells, macrophages, and degenerative or necrotic cells found in the edges or centres of the tubercles. There was less-sufficient infiltration of lymphocytes. Most lung sections from three Group-1 macaques (Drug alone) exhibited histopathology of haemorrhages (red arrows) or necrotic granulomas (black arrows), although less necrotic or focal granulomas were also seen. In contrast, ZOL/IL2-treated (Group-2) and IL2-treated (Group-3) macaques generally showed small non-necrotic or less-necrotic granulomas, and localized or contained by large numbers of lymphocytes as pointed by small black arrows. The unbiased processes for collecting tissues for histopathology were performed as we previously described [7,22].

**Figure:**
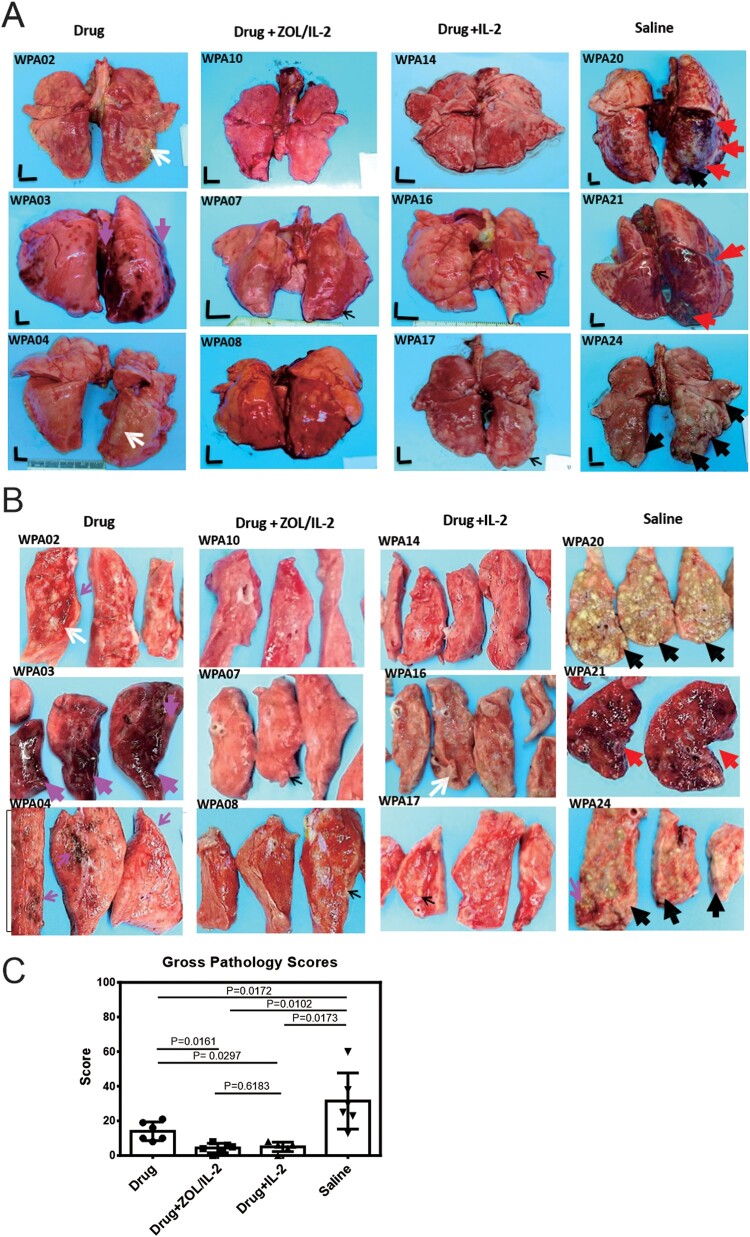

**Figure:**
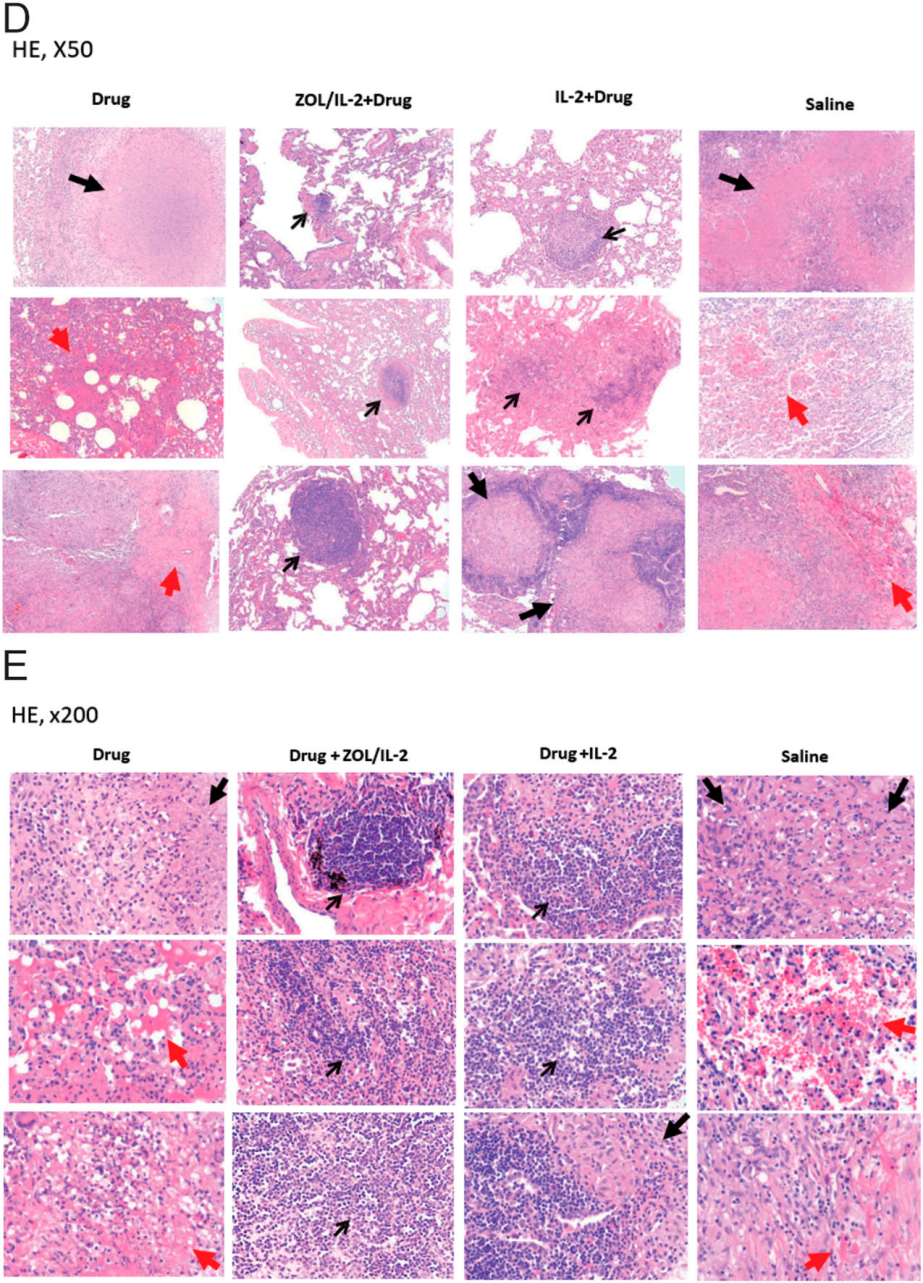

When the entire TB pathology was compared between groups using established quantitative scoring criteria [7,22,32], the comparison confirmed that the ZOL/IL-2-treated (Group-2) and IL-2-treated (Group-3) macaques had significantly milder TB lesions or pathology than the TB drugs alone and saline control groups (Figure 7(C); *p* < 0.05, respectively). Overall, the macroscopic MDR-TB pathology lesions were consistent with the histopathological changes in lung sections (Figure 7(D,E)). Virtually, the lung sections (Figure 7(D,E)) from the saline group of macaques exhibited severe TB lesions (large black arrows) and different stages of haemorrhagic changes (large red arrows). The recent haemorrhages displayed residual erythrocytes/debris or pigment haemoglobin; earlier haemorrhage appeared to be predominated by post-haemorrhage necrosis or destruction. The severe TB lesions were characterized by widespread necrosis and tissue destruction. Most lung sections from three Group-1 macaques (TB Drug alone) exhibited histopathology of haemorrhages (red arrows) or necrotic granulomas (black arrows), although less necrotic or focal granulomas were also seen. In contrast, ZOL/IL2-treated (Group-2) and IL2-treated (Group-3) macaques generally showed small non-necrotic or less-necrotic granulomas, and localized or contained by large numbers of lymphocytes as pointed by small black arrows (Figure 7(D,E)).

Together, the results above suggest that adjunctive ZOL/IL-2 (Group-2) or IL-2 (Group-3) administrations after MDR-TB infection and TB drug treatment led to milder MDR-TB pathology/lesions than TB drug alone and saline controls. However, ZOL/IL-2 administrations led to significantly-lower MDR-TB infection burdens than IL-2 alone.

## Discussion

In the current study, we found that adjunctive ZOL/IL-2 administrations during TB drug treatment of MDR-Mtb-infected macaques led to significantly lower levels of MDR-TB bacterial burdens than adjunctive IL-2, TB drugs alone, and saline controls, respectively, with favourable clinical outcomes as body weights gains. While adjunctive ZOL/IL-2 regimen clearly could result in milder MDR-TB pathology than TB drugs alone or saline controls, there was no significant difference in TB pathology between ZOL/IL-2-treated and IL-2-treated macaques. This was not totally unexpected. Published studies from us and others demonstrated that IL-2 alone could exert immunotherapeutics against TB infection and TB lesions [8,33,34], whereas greater expansions of Vγ2Vδ2 T effector cells by more potent Picostim (HMBPP analog) plus IL2 could control primary TB infection better than IL2 alone [7].

Adjunctive ZOL/IL2 immunotherapeutic effects correlate with ZOL/IL2-induced early massive expansion of Vγ2Vδ2 T cells, rapid/sustained increases in Vγ2Vδ2 T effector cells producing anti-TB cytokines IFN-γ/TNF-α/perforin during the almost entire course of treatments. Consistently, ZOL/IL-2-expanded Vγ2Vδ2 T cells could potently inhibit the growth of mycobacteria in host cells. Each of the above cytokines contributes to Vγ2Vδ2 T cell-mediated inhibition of TB bacilli growth [7]. Notably, the adjunctive ZOL/IL-2 therapeutic efficacy also correlated with its ability to facilitate or enhance faster and sustained immune responses of CD4^+^ Th1, CD8^+^ T effectors, and Foxp3^+^ Treg cells. While CD4^+^ Th1 and CD8^+^ T effector cells have been reported to reproducibly control high-dose TB infection in NHP, we and others also showed that IL-2-induced Foxp3^+^ Treg can have both the inflammation-suppression and the inhibitory effects on intracellular *Mtb* in macrophages [7,15,21,35–37]. Thus, Foxp3^+^ T cells induced by IL2 or ZOL + IL2 regimen in primary MDR-TB infection appear to be anti-TB, rather than simply immune-suppressive, and such IL2-induced Foxp3^+^ T cells might be different from conventional Foxp3^+^ Treg cells generated during chronic TB disease. Together, protective Vγ2Vδ2 T effector cells, CD4^+^ Th1, CD8^+^ T effectors, and Foxp3^+^ Treg, along with their production of anti-TB cytokines, may serve as the mechanisms underlying adjunctive ZOL/IL2 therapeutics against MDR-TB.

ZOL + IL2 treatment would similarly increase the frequency and function of Vγ2Vδ2 T cells in lung tissues while they expanded in the airway BAL and the circulation as we previously demonstrated [7]. A lack of *in-situ* lung-tissue data was related to the fact that due to more stringent biosafety protocols for MDR-TB-infected macaques, we were unable to take fresh lung tissues out of ABSLIII facilities for in-depth experiments (convincing data could readily be generated from sections derived from fresh lungs, but not formalin-fixed tissues). However, adjunctive administrations of ZOL/IL2 in Group-2 macaques readily increased numbers of some anti-TB γδ and αβ T effector populations in airway BAL fluid at week 3 until week 19 or 21 after MDR-TB infection, although entire T effector cells in airway were less consistently detected overtime. These less-consistent changes might be explained by the activation-induced T-cell homeostasis/exhaustion or by a potential accumulation of those effector cells in lung mucosae as detected in our previous study [22], rather than in airway BAL fluid. The potential residence of protective γδ and αβ T effector cells in lung tissues was also supported by our observation that IFN-γ-producing Vγ2Vδ2 T effector cells, CD4^+^ Th1 and CD8^+^ T effectors including CD25^+^ effectors were detected in the circulation at higher levels during the almost entire ZOL/IL-2 intervention of MDR-TB infection.

To optimally uncover potential differences in therapeutic effects, we employed two useful strategies (intermittent TB drugs and Adalimumab) during the treatment of MDR-TB V791 infection of macaques. In the first strategy, we adopted the intermittent TB drug treatment, rather than daily treatment for the entire ∼4 months of treatment. This experimental design was based on the following clinical principles and publications: (i) fresh or primary MDR-TB V791 infection of macaques were more readily controlled by sensitive antibiotics treatment than the chronically-evolved MDR-TB in patients who are refractory or resistant to treatments; (ii) intermittent TB drug treatments would avoid the ability of TB drugs to overshadow or cover potential immunotherapeutic effects [12]. It has been reported that adjunctive IL2 treatment of drug-sensitive TB patients in combination with multiple sensitive anti-TB drugs (antibiotics) failed to uncover IL2 therapeutic effect [12], despite that other studies clearly demonstrated that IL2 can control TB infection [8]. Sensitive TB drugs are able to control TB infection rapidly and more effectively than immunotherapeutic, because sensitive anti-TB antibiotics presumably can directly kill extracellular and intracellular TB bacilli, and because immune drugs indirectly act on T cells with no or less killing of TB bacilli [8]. Here, our intermittent TB drugs treatments controlled MDR-TB compared to saline control C (Figure 6(A) CFU, Figure 7(C) Pathology score), but appeared to result in a residual or subclinical MDR-TB infection. Such optimal setting allowed us to demonstrate that adjunctive ZOL/IL2 or IL2 therapeutics led to better control MDR-TB infection burdens and TB lesions than TB drugs alone regimen.

Using another useful experimental strategy, we employed Adalimumab treatment to activate or enhance subclinical or inactive MDR-TB infection [13] for optimal comparisons between test and control groups at endpoints. Interestingly, Adalimumab enhancing of MDR-TB V791 infection uncovered TB-associated haemorrhages in TB drugs alone (Group-1) and saline (Group-4) controls. The MDR-TB haemorrhages here appear to be consistent with TB-associated haemorrhages manifested as haemoptysis in TB patients [38].

In summary, our results suggest that Vγ2Vδ2 T cells that exist only in humans and NHP are promising host-directed targets for anti-TB immunotherapeutics. Because both ZOL and IL-2 have widely been used in clinics for treatments of human diseases, adjunctive ZOL/IL-2 administration in combination with anti-TB drugs is the attractive immune regimen for treatments of MDR-TB. The findings in the current study prove the concept and support the future efforts of moving forward to test adjunctive ZOL/IL-2 immunotherapeutic against MDR-TB in humans.

## Supplementary Material

Supplemental Material

## References

[1] Organization GWH. WHO Treatment Guidelines for Drug-Resistant Tuberculosis, 2016 Update. 2016. 27748093

[2] Chung-Delgado K, Guillen-Bravo S, Revilla-Montag A, et al. Mortality among MDR-TB cases: comparison with drug-susceptible tuberculosis and associated factors. PLoS One. 2015;10(3):e0119332. doi:10.1371/journal.pone.0119332 25790076 PMC4366185

[3] Kilinç G, Saris A, Ottenhoff THM, et al. Host-directed therapy to combat mycobacterial infections. Immunol Rev 2021;301(1):62–83. doi:10.1111/imr.12951 33565103 PMC8248113

[4] Young C, Walzl G, Plessis ND. Therapeutic host-directed strategies to improve outcome in tuberculosis. Mucosal Immunol 2020;13(2):190–204. doi:10.1038/s41385-019-0226-5 31772320 PMC7039813

[5] Shen Y, Zhou D, Qiu L, et al. Adaptive immune response of Vgamma2Vdelta2+ T cells during mycobacterial infections. Science. 2002;295(5563):2255–2258. doi:10.1126/science.1068819 11910108 PMC2872146

[6] Qaqish A, Huang D, Chen CY, et al. Adoptive transfer of phosphoantigen-specific γδ T cell subset attenuates *Mycobacterium tuberculosis* infection in nonhuman primates. J Immunol 2017;198(12):4753–4763. doi:10.4049/jimmunol.1602019 28526681 PMC5557270

[7] Chen CY, Yao S, Huang D, et al. Phosphoantigen/IL2 expansion and differentiation of Vγ2Vδ2 T cells increase resistance to tuberculosis in nonhuman primates. PLoS Pathog. 2013;9(8):e1003501. doi:10.1371/journal.ppat.1003501 23966854 PMC3744401

[8] Chen CY, Huang D, Yao S, et al. IL-2 simultaneously expands Foxp3+ T regulatory and T effector cells and confers resistance to severe tuberculosis (TB): implicative Treg-T effector cooperation in immunity to TB. J Immunol 2012;188(9):4278–4288. doi:10.4049/jimmunol.1101291 22474020 PMC3412415

[9] Meraviglia S, Eberl M, Vermijlen D, et al. In vivo manipulation of Vγ9Vδ2 T cells with zoledronate and low-dose interleukin-2 for immunotherapy of advanced breast cancer patients. Clin Exp Immunol 2010;161(2):290–297. doi:10.1111/j.1365-2249.2010.04167.x 20491785 PMC2909411

[10] Yang E, Yang R, Guo M, et al. Multidrug-resistant tuberculosis (MDR-TB) strain infection in macaques results in high bacilli burdens in airways, driving broad innate/adaptive immune responses. Emerg Microbes Infect 2018;7(1):207. 30538219 10.1038/s41426-018-0213-z PMC6290002

[11] Wang F, Shao L, Fan X, et al. Evolution and transmission patterns of extensively drug-resistant tuberculosis in China. Antimicrob Agents Chemother 2015;59(2):818–825. doi:10.1128/AAC.03504-14 25403663 PMC4335892

[12] Johnson JL, Ssekasanvu E, Okwera A, et al. Randomized trial of adjunctive interleukin-2 in adults with pulmonary tuberculosis. Am J Respir Crit Care Med 2003;168(2):185–191. doi:10.1164/rccm.200211-1359OC 12702550

[13] Lin PL, Maiello P, Gideon HP, et al. PET CT identifies reactivation risk in cynomolgus macaques with latent M. tuberculosis. PLoS Pathog 2016;12(7):e1005739. doi:10.1371/journal.ppat.1005739 27379816 PMC4933353

[14] Ryan-Payseur B, Frencher J, Shen L, et al. Multieffector-functional immune responses of HMBPP-specific Vγ2Vδ2 T cells in nonhuman primates inoculated with Listeria monocytogenes ΔactA prfA*. J Immunol 2012;189(3):1285–1293. doi:10.4049/jimmunol.1200641 22745375 PMC3412419

[15] Chen CY, Huang D, Wang RC, et al. A critical role for CD8 T cells in a nonhuman primate model of tuberculosis. PLoS Pathog 2009;5(4):e1000392. doi:10.1371/journal.ppat.1000392 19381260 PMC2663842

[16] Shen H, Wang Y, Chen CY, et al. Th17-related cytokines contribute to recall-like expansion/effector function of HMBPP-specific Vγ2Vδ2 T cells after *Mycobacterium tuberculosis* infection or vaccination. Eur J Immunol 2015;45(2):442–451. doi:10.1002/eji.201444635 25141829 PMC4916493

[17] Yang R, Yao L, Shen L, et al. IL-12 expands and differentiates human Vγ2Vδ2 T effector cells producing antimicrobial cytokines and inhibiting intracellular mycobacterial growth. Front Immunol 2019;10(913):1–13. 31080452 10.3389/fimmu.2019.00913 PMC6497761

[18] Yang R, Yang E, Shen L, et al. IL-12+IL-18 cosignaling in human macrophages and lung epithelial cells activates cathelicidin and autophagy, inhibiting intracellular mycobacterial growth. J Immunol 2018;200(7):2405–2417. doi:10.4049/jimmunol.1701073 29453279 PMC5860987

[19] Yang R, Peng Y, Pi J, et al. A CD4+CD161+ T-cell subset present in unexposed humans, Not Tb patients, are fast acting cells that inhibit the growth of intracellular mycobacteria involving CD161 pathway, perforin, and IFN-γ/autophagy. Front Immunol 2021;12:599641. doi:10.3389/fimmu.2021.599641 33732233 PMC7959736

[20] Shen H, Gu J, Liang S, et al. Selective destruction of IL-23 induced expansion of major antigen-specific γδ T-cell subset in TB patients. J Infect Dis 2017;215(3):420–430. 27789724 10.1093/infdis/jiw511 PMC5853380

[21] Yao S, Huang D, Chen CY, et al. CD4+ t cells contain early extrapulmonary tuberculosis (TB) dissemination and rapid TB progression and sustain multieffector functions of CD8+ T and CD3- lymphocytes: mechanisms of CD4+ T cell immunity. J Immunol 2014;192(5):2120–2132. doi:10.4049/jimmunol.1301373 24489088 PMC4104690

[22] Shen L, Frencher J, Huang D, et al. Immunization of Vγ2Vδ2 T cells programs sustained effector memory responses that control tuberculosis in nonhuman primates. Proc Natl Acad Sci U S A. 2019;116(13):6371–6378. doi:10.1073/pnas.1811380116 30850538 PMC6442559

[23] Wang F, Huang G, Shen L, et al. Genetics and functional mechanisms of STAT3 polymorphisms in human tuberculosis. Front Cell Infect Microbiol 2021;11:669394. doi:10.3389/fcimb.2021.669394 34307193 PMC8294188

[24] Huang D, Chen CY, Ali Z, et al. Antigen-specific Vgamma2Vdelta2 T effector cells confer homeostatic protection against pneumonic plaque lesions. Proc Natl Acad Sci U S A. 2009;106(18):7553–7558. doi:10.1073/pnas.0811250106 19383786 PMC2678605

[25] Shen L, Huang D, Qaqish A, et al. Fast-acting γδ T-cell subpopulation and protective immunity against infections. Immunol Rev 2020;298(1):254–263. doi:10.1111/imr.12927 33037700

[26] Brandes M, Willimann K, Bioley G, et al. Cross-presenting human γδ T cells induce robust CD8+ αβ T cell responses. Proc Natl Acad Sci U S A. 2009;106(7):2307–2312. doi:10.1073/pnas.0810059106 19171897 PMC2650152

[27] Zorn E, Nelson EA, Mohseni M, et al. IL-2 regulates FOXP3 expression in human CD4+CD25+ regulatory T cells through a STAT-dependent mechanism and induces the expansion of these cells in vivo. Blood 2006;108(5):1571–1579. doi:10.1182/blood-2006-02-004747 16645171 PMC1895505

[28] Boyman O, Sprent J. The role of interleukin-2 during homeostasis and activation of the immune system. Nat Rev Immunol 2012;12:180–190. doi:10.1038/nri3156 22343569

[29] Sarhan D, Leijonhufvud C, Murray S, et al. Zoledronic acid inhibits NFAT and IL-2 signaling pathways in regulatory T cells and diminishes their suppressive function in patients with metastatic cancer. Oncoimmunology 2017;6(8):e1338238. doi:10.1080/2162402X.2017.1338238 28920001 PMC5593706

[30] Triplett TA, Curti BD, Bonafede PR, et al. Defining a functionally distinct subset of human memory CD4+ T cells that are CD25POS and FOXP3NEG. Eur J Immunol 2012;42(7):1893–1905. doi:10.1002/eji.201242444 22585674

[31] Seedat UF, Seedat F. Post-primary pulmonary TB haemoptysis – when there is more than meets the eye. Respir Med Case Rep. 2018;25:96–99. 30094156 10.1016/j.rmcr.2018.07.006 PMC6080505

[32] Lin PL, Dietrich J, Tan E, et al. The multistage vaccine H56 boosts the effects of BCG to protect cynomolgus macaques against active tuberculosis and reactivation of latent *Mycobacterium tuberculosis* infection. J Clin Invest 2012;122(1):303–314. doi:10.1172/JCI46252 22133873 PMC3248283

[33] Liu X, Li F, Niu H, et al. IL-2 Restores T-cell dysfunction induced by persistent *Mycobacterium tuberculosis* antigen stimulation. Front Immunol 2019;10:2350. doi:10.3389/fimmu.2019.02350 31632413 PMC6783502

[34] Zhang R, Xi X, Wang C, et al. Therapeutic effects of recombinant human interleukin 2 as adjunctive immunotherapy against tuberculosis: a systematic review and meta-analysis. PLoS One 2018;13(7):e0201025. doi:10.1371/journal.pone.0201025 30024982 PMC6053227

[35] Cardona P, Cardona P-J. Regulatory T cells in *Mycobacterium tuberculosis* infection. Front Immunol 2019;10(2139):1–11. 31572365 10.3389/fimmu.2019.02139 PMC6749097

[36] Kauffman KD, Sallin MA, Sakai S, et al. Defective positioning in granulomas but not lung-homing limits CD4 T-cell interactions with *Mycobacterium tuberculosis*-infected macrophages in rhesus macaques. Mucosal Immunol 2018;11(2):462–473. doi:10.1038/mi.2017.60 28745326 PMC5785573

[37] Sharan R, Singh DK, Rengarajan J, et al. Characterizing early T cell responses in nonhuman primate model of tuberculosis. Front Immunol 2021;12(706723):1–9. 10.3389/fimmu.2021.706723 PMC8416058 34484203

[38] Seedat UF, Seedat F. Post-primary pulmonary TB haemoptysis – when there is more than meets the eye. Respir Med Case Rep 2018;25:96–99. 30094156 10.1016/j.rmcr.2018.07.006 PMC6080505

