# Supplementary material for: Adjunctive Zoledronate + IL-2 administrations enhance anti-tuberculosis Vγ2Vδ2 T-effector populations, and improve treatment outcome of multidrug-resistant tuberculosis1
Source: Emerg Microbes Infect. 2022 Jul 21;11(1):1790–805. doi: 10.1080/22221751.2022.2095930 (PMC9310823; doi:10.1080/22221751.2022.2095930)
Supplement: Supplemental Material [file TEMI_A_2095930_SM8289.docx]

**Supplemental Table. Drugs used in this study.**

| **Drugs Name** | **size** | **mg/kg** | **Company** | **Administration** |
| --- | --- | --- | --- | --- |
| Ethambutol (E) | 0.25g/tablet | 0.017g/kg | Guangdong Taicheng Pharmaceutical Co.LTD | po |
| Avelox(Moxifloxacin, Mfx) | 0.4g/tablet | 0.01g/kg | Bayer Pharma AG | po |
| Capreomycin sulfate for injection (CM) | 0.75g/dose | 0.02g/kg | Zhejiang Hisun Pharmaceutical Co.LTD | im |
| ZOMETA (Zoledronic Acid, Zol) | 4mg/dose | 0.06 mg/kg | Novartis Pharma Stein AG | iv |
| QUANQI(Recombinant Human Interleukin-2(I) for Injection, IL-2) | 1million U/dose | 0.25 million U/kg | Shandong Quangang Pharmaceutical Co.LTD | id |
| Adalimumab | 40mg/0.8ml | 2.5mg/kg | ABBOTT, IL | subcutaneous |
